# Identification of stable reference genes for qRT-PCR in *Stropharia rugosoannulata* using mRNA-sequencing data

**DOI:** 10.1371/journal.pone.0323272

**Published:** 2025-05-07

**Authors:** Xiaohua Zhang, Xuesong Li, Rong Hua, Yuan Fang, Tingsong Yue, Jianying Li, Yuxun Lu, Wansong Yue, Zhanghui Gao, Shaoxiong Liu, Dafeng Sun

**Affiliations:** 1 Kunming Edible Fungi Institute of All China Federation of Supply and Marketing Cooperatives, Kunming, China; 2 Yunnan Academy of Edible Fungi Industry Development, Kunming, China; 3 College of Biological and Chemical Engineering, Guangxi University of Science and Technology, Liuzhou, China; ICAR-Directorate of Mushroom Research, INDIA

## Abstract

Quantitative real-time PCR (qRT-PCR) is a well-established and reliable technology utilized for the rapid and accurate quantification of gene expression changes. The selection of stable reference genes is necessary to analyse qRT-PCR data and ensure gene expression studies reliability. *Stropharia rugosoannulata*, commonly known as the wine-cap *Stropharia* mushroom, ranks among the top ten internationally traded mushrooms. In the present study, six novel candidate reference genes were selected from *S. rugosoannulata* transcriptome, alongside four traditional reference genes that displayed stable expression levels in *S. rugosoannulata*. Three widely used software (geNorm, NormFinder, and BestKeeper) were employed to analyse ten candidate reference genes, and the final ranking of reference genes was determined through RefFinder. The results indicated that *UBP* exhibited the highest stability across various developmental stages of red and yellow *S. rugosoannulata*, while *RPB2* and *GAPDH* showed the least stability. These novel reference genes demonstrated significantly superior stability to other four traditional genes across nearly all developmental stages. In conclusion, Our findings provide robust guidelines for selecting suitable reference genes, thereby enhancing the reliability of qRT-PCR normalization in *Stropharia rugosoannulata*.

## Introduction

*Stropharia rugosoannulata*, commonly referred to as the wine-cap *Stropharia* mushroom, is a popular edible mushroom with high medicinal and nutritional values [1]. Therefore, the Food and Agriculture Organization (FAO) has recommended *S. rugosoannulata* as a valuable food source for developing countries [2]. Its uncomplicated cultivation requirements and impressive stress tolerance enable it to thrive on various agricultural waste materials, including straw, corncobs, wood chips, sawdust, and rice husks, yielding substantial economic benefits. These characteristics establish *S. rugosoannulata* as a promising edible mushroom in China. Moreover, this species is nutritionally rich, containing all essential amino acids and several non-essential amino acids that are beneficial to human health [3]. *S. rugosoannulata* was first introduced to China from Poland in the 1980s and successfully cultivated, although it was not promoted at the time. In recent years, advancements in cultivation techniques and the growing emphasis on sustainable agricultural practices, particularly the microbial degradation of straw, have significantly increased the popularity of *S. rugosoannulata* cultivation in China [4]. By 2019, field cultivation of *S. rugosoannulata* had expanded to most provinces across the country, with an estimated cultivation area reaching approximately 1,333 hectares [5]

Gene expression analysis is vital for elucidating gene functions, and examining gene expression patterns can provide significant insights into these functions [6]. Various methodologies, such as northern blotting, quantitative real-time reverse transcription polymerase chain reaction (qRT-PCR), gene chips [7], and RNA sequencing (RNA-seq) [8], are employed in gene expression studies. Among these methods, qRT-PCR has emerged as a prominent method for the rapid and precise evaluation of gene expression changes to elucidate gene functions and regulatory mechanisms associated with specific scientific questions [9–11]. However, numerous factors—including initial RNA quality, reverse transcription efficacy, primer specificity, PCR conditions, amplification efficiency, and transcript normalization—can significantly impact the reliability of qRT-PCR results [12–16]. Notably, the normalization of qRT-PCR data using appropriate internal reference genes is crucial for minimizing technical variability [13,14], while unsuitable reference genes can lead to misleading conclusions [17,18]. Recent findings suggest that there is no universal reference gene applicable across all species; in fact, optimal reference genes may vary considerably even within the same species, depending on developmental stages or treatment conditions [19,20]. Therefore, selecting appropriate reference genes tailored to specific experimental conditions and species is imperative [21,22].

The genome of *S. rugosoannulata* was first reported in 2022 [23]. The completion of its whole genome has catalyzed significant research in identifying specific functional genes, the mechanisms of lignin and cellulose degradation, and the biosynthesis of metabolites [5]. Recent transcriptomic analyses have explored differential gene expression patterns during the developmental stages of *S. rugosoannulata* [4,22]. Furthermore, comprehensive transcriptome profiling has been employed to investigate the molecular mechanisms underlying flavonoid biosynthesis [24], carbohydrate metabolism under low-temperature conditions [4].However, the findings derived from these transcriptomic analyses remain to be experimentally validated at the gene expression level. The accurate quantification of gene expression profiles in *S. rugosoannulata* is necessitates the identification and application of appropriate reference genes. Consequently, the systematic identification and validation of reliable reference genes in *S. rugosoannulata* represents a critical research priority, which would provide crucial tools for elucidating its growth regulation, developmental mechanisms, and fundamental biological characteristics.

Research on the selection and evaluation of reference genes has been conducted in various mushroom species such as *Lentinula edodes* [12], *Ganoderma lucidum* [25], and *Pleurotus ostreatus* [26]. However, these studies have predominantly relied on traditional reference genes derived from prior studies in plants or animals, such as actin, tubulin, and *18S rRNA*. Due to inherent species differences and distinct regulatory mechanisms, these traditionally reference genes may not be suitable for gene expression analysis in mushrooms. For instance, the commonly used reference gene *18S rRNA* has been deemed inappropriate for *Volvariella volvacea* [20], *Ganoderma lucidum* [25], and *Auricularia cornea* [19]. Therefore, there is an urgent need to systematically re-evaluate and identify novel reference genes suitable for *S. rugosoannulata*.

Several studies have successfully screened candidate reference genes from transcriptomic data in plants [27], animals [28], and fungi [29]. Similar approaches are increasingly being applied to mushroom species, suggesting the feasibility and accuracy of the methodology [20,30]. Nonetheless, research focusing on reference genes for qRT-PCR normalization from transcriptomic data in *S. rugosoannulata* remains scarce, hindering the analysis of target gene expression across various growth stages or cultivation conditions. The advent of omics technologies and the publication of the *S. rugosoannulata* genome and transcriptome present an opportunity to identify and validate reliable reference genes for qRT-PCR normalization [5].

In this study, we identified six candidate reference genes from mRNA sequencing data (unpublished) of yellow and red *S. rugosoannulata* across critical developmental stages, from mycelium to the fruiting body. Additionally, we included four traditional reference genes previously reported to exhibit relatively stable expression in this species [31]. The expression levels of these candidate reference genes were further assessed using qRT-PCR alongside statistical software such as geNorm [32], NormFinder [33], and BestKeeper [34], complemented by RefFinder [35] for comprehensive stability assessment. The outcomes of this research will provide valuable insights for reference gene selection in gene expression studies and contribute to establishing a reliable set of reference genes for qRT-PCR normalization in *S. rugosoannulata*.

## Materials and methods

### Sample preparation and culture conditions

For this study, six samples were selected, encompassing mycelium, primordia, and fruiting bodies of both yellow and red *S. rugosoannulata*. The yellow strain (ZJQGG001) and the red strain were sourced from the Kunming Edible Fungi Institute of the All-China Federation of Supply and Marketing Cooperatives. Cultures were established on potato dextrose agar (PDA), incubated at 25°C for 7 days. Primordia and fruiting bodies were harvested from Kunming, Yunnan Province, China. The substrate for cultivation consisted of 42% sawdust, 30% rice husk, 5% wheat bran, 1% gypsum, 1% calcium superphosphate, and 20% crushed corncobs (particle size: 0.5 cm–1 cm), with moisture levels adjusted to 55%-65%. All samples were subsequently frozen in liquid nitrogen and stored at -80°C until RNA extraction.

### Total RNA extraction and cDNA synthesis

Total RNA was extracted from each sample utilizing the Takara MiniBEST Universal RNA Extraction Kit (Takara, Shiga, Japan) following the manufacturer’s instructions. RNA was diluted in RNase-free water, and the purity and concentration were assessed with a NanoDrop 2000 Spectrophotometer (NanoDrop Technologies, Thermo Scientific, USA). RNA integrity was confirmed by electrophoresis on a 1.5% (w/v) agarose gel. Only RNA samples meeting the criteria of A260/280 ratios between 1.8 and 2.2 and A260/230 ratios above 1.8 were employed for cDNA synthesis. One microgram of total RNA was reverse-transcribed into 20 μL cDNA using random primers and an oligo dT primer with the PrimeScript™ RT Reagent Kit (with gDNA Eraser) (Takara, Shiga, Japan). The synthesized cDNA was diluted fivefold with nuclease-free water and stored at -20°C.

### Primer design and amplification by qRT-PCR

Primers were designed using Primer3 software (http://primer3.ut.ee/) based on the genomic data of *S. rugosoannulata*, adhering to specific design criteria: a length of 20–27 bp, GC content of 45–55%, a melting temperature (Tm) range of 55–60°C, and amplicon sizes between 140–200 bp. The primers were synthesized by Tsingke, and detailed information is provided in Table 1. The specificity of each qRT-PCR reaction was confirmed through melting curve analysis (temperature gradient from 60 to 95°C). Standard qRT-PCR was conducted for all primer pairs, and the presence of a single PCR product was verified via electrophoresis on a 1.5% agarose gel.

**Table 1:** Candidate genes, primers, and different parameters derived from the qRT-PCR analysis.

Gene	Description	Products length(bp)	Primer sequence((5′ → 3′)	Efficiency (%)	R^2^
*LEO1*	RNA polymerase-associated protein LEO1	175	F:CATCAAGCTCAAGGTCGAAAACA R:TATCGTGGCAGAGGTATCAATGG	102.01	0.998
*UBP*	Oligouridylate-binding protein	123	F:CTCAACAACCTGGCTCTGATCT R:GCTGTTTGTGCTCCGTAATACC	91.65	0.999
*MTC*	Maintenance of telomere capping protein	141	F:TCTCAATGTTGTGGCCCCTC R:TCGCCTCCCTTCACTTGTTC	103.52	0.997
*CKA1*	Casein kinase II subunit alpha	118	F:TATGAGATTGTACGGAAAGTGGGG R:TCTTCTTCTTCTTCACTGGCTTCA	107.63	0.989
*VMA*	V type (H+) ATPase V1	170	F:ATCAGGGATGAGGAACGGGA R:TGGATGGTGGCTTTGTCTCC	93.83	0.998
*MRP*	Mitochondrial protein	139	F:GCGTAGTTATTCTTTTAGCGGTGG R:TACGTTAGGATAGATGTCGGCAAG	90.69	0.996
*RPB2*	DNA-direct RNA polymerase subunit 2	245	F:AACCCTCGCCAATAAACTTCCT R:TTGGGTGTCCTGCATGTCATAA	103.95	0.964
*Actin*	Actin-related protein 2	150	F:GGAAATGAAGCAACTCCATCTGG R:TCACGGTTTTTCATAATGTCGGC	100.5	0.996
*UBQ*	polyubiquitin	179	F:CAACGTCAAGGCCAAAATCCA R:CCTTCAGCGAGGGTTCAATGA	101.45	0.999
*GAPDH*	glyceraldehyde-3-phosphate dehydrogenase	141	F:ATGCCCCCATGTTCGTCTG R:AGCCCTCAACAATGCCGAA	96	0.999

Real-time amplification reactions were executed using the LineGene9600 (Bioer Technology, China) system. Each reaction was composed of a total volume of 20 μL, including 2 μL of fivefold diluted cDNA template, 0.8 μL of each amplification primer (10 μM), 10 μL of TB Green Premix Ex Taq™ II (Takara, Shiga, Japan), and nuclease-free water for volume adjustment. The amplification conditions entailed an initial denaturation at 95°C for 40 seconds, followed by 40 cycles of denaturation at 95°C for 10 seconds and annealing/extension at 60°C for 15 seconds. Triplicate analyses were conducted for each biological sample.

To ascertain amplification efficiency (E) and correlation coefficients (R² values) for each primer pair, standard curves were generated using tenfold serial dilutions of cDNA. R² values and amplification efficiencies (E) for each primer pair were calculated from the regression line slope by plotting mean Cq values against the log cDNA dilution factor in Microsoft Excel, using the equation E (%) = (10^(-1/slope)−1)×100.

### Data analysis

To identify suitable reference genes, the expression stability of candidate reference genes was assessed using geNorm, NormFinder, BestKeeper, and the web-based tool RefFinder. RefFinder consolidates and ranks all candidate reference genes by assigning an appropriate weight to each gene based on their stability. For geNorm and NormFinder analyses, raw Ct values were converted into relative quantities using the formula 2^(-ΔCt), where ΔCt is defined as the difference between the Ct value of each sample and the lowest Ct value for the same gene across different samples. In contrast, BestKeeper and RefFinder analyses utilized the untransformed Ct values as input data. The expression stability of the candidate genes was evaluated by integrating the results obtained from these software tools.

## Results

### Screening and identification of candidate reference genes

**Fig 1:**
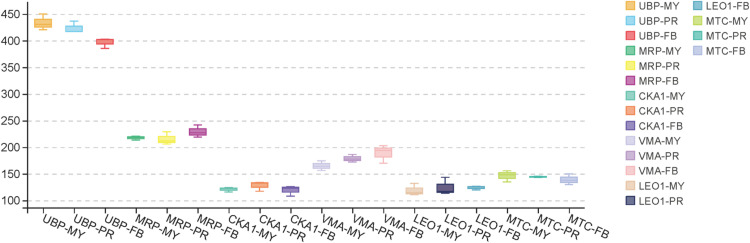
The FPKM values for the novel candidate genes at various developmental stages. The boxes depict the 25/75 percentiles; Lines across the boxes represent the medians; Error bars represent the maximum and minimum FPKM values.MY: mycelium; PR: primordial; FB: fruiting bodies.

In this investigation, ten candidate reference genes were selected for expression stability analysis, with six being evaluated for the first time. These novel genes were identified from mRNA sequencing data of red and yellow *S. rugosoannulata* across three developmental stages, based on their expression levels quantified as fragments per kilobase million (FPKM). The coefficient of variation and standard deviation (SD) for FPKM were computed across the dataset to pinpoint genes with relatively low coefficients as candidates. The selected genes included Oligouridylate-binding protein (*UBP*), Maintenance of telomere capping protein (*MTC*), RNA polymerase-associated protein LEO1 (*LEO1*), Casein kinase II subunit alpha (*CKA1*), Pre-mRNA-processing protein (*PRP*), and V type (H+) ATPase V1 (*VMA*). Additionally, four commonly used reference genes—glyceraldehyde-3-phosphate dehydrogenase (*GAPDH*), polyubiquitin (*UBQ*), Actin-related protein 2 (*ACTIN*), and DNA-directed RNA polymerase subunit 2 (*RPB2*)—demonstrated reliability in preliminary studies involving *S. rugosoannulata*. The FPKM values for the novel candidate genes at various developmental stages are shown in Fig 1. These candidate genes exhibited greater stability compared to traditional reference genes across developmental stages.

### Expression profiles of candidate reference genes

**Fig 2:**
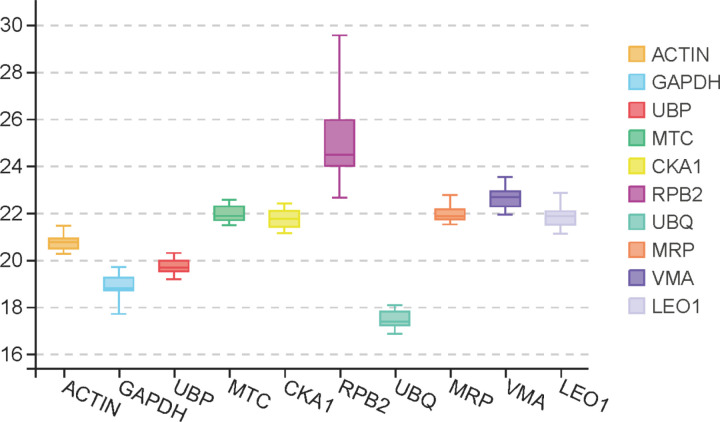
The range of CP values of 10 reference gene candidates for all samples. The boxes depict the 25/75 percentiles; Lines across the boxes represent the medians; Error bars represent the maximum and minimum Cp values. The 10 candidate reference genes are listed on the x-axis.

The mean cycle threshold (Ct) values of ten candidate reference genes were assessed to determine their expression stability across all experimental samples. The expression profiles are illustrated in Fig 2, showing mean Ct values ranging from 17.4 to 25.1, predominantly between 18 and 23. *UBP* demonstrated the least variation (19.1 to 20.2), indicating its potential as an optimal reference gene, while *RPB2* exhibited the highest variation (22.6 to 29.6), suggesting it should be excluded as a reference gene. The variability in Ct values across samples emphasizes the necessity of evaluating the expression stability of these candidate genes.

### Reference gene stability analysis

**Fig 3:**
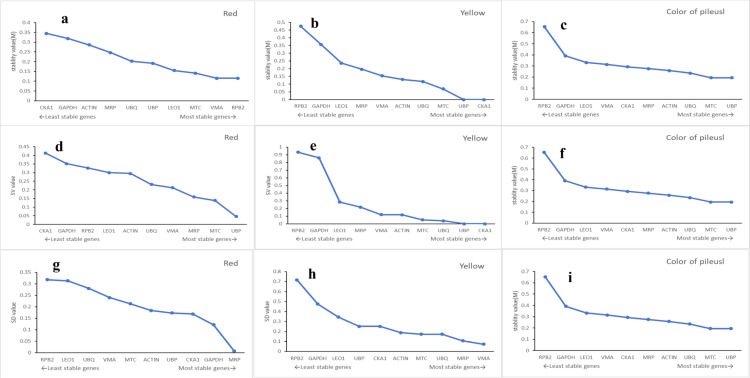
Rankings of candidate reference genes for *S. rugosoannulata* with yellow, red, and different colors of the pileus as evaluated by geNorm (a-c), BestKeeper (d-f), and NormFinder (g-i).

Using geNorm analysis, the expression stability (M values) was calculated based on pairwise variation among reference genes. Lower M values reflect higher stability. In yellow *S. rugosoannulata*, *UBP* exhibited the lowest M value, making it the most stable gene, followed by *CKA1*; conversely, *RPB2* was the least stable. For red *S. rugosoannulata*, *VMA* and *RPB2* were the most stable genes, while *CKA1* exhibited the least stability (Fig 3).

Additionally, NormFinder was employed to assess expression stability by calculating inter- and intra-group variability, assigning stability values (SV) to each reference gene. Lower SV values indicate greater stability. In yellow *S. rugosoannulata*, *UBP* and *CKA1* were identified as the most stable genes, with *RPB2* being the least stable. In red samples, *UBP* and *MTC* were identified as the most stable genes, with *CKA1* being the least stable (Fig 3).

Both geNorm and NormFinder yielded consistent results, identifying *UBP* and *CKA1* as the most stable genes, and *RPB2* as the least stable gene in yellow *S. rugosoannulata*. However, there were discrepancies in red *S. rugosoannulata*, with geNorm ranking *VMA* and *RPB2* as the most stable, while NormFinder favored *UBP* and *MTC* (Fig 3). Thus, further analysis was warranted.

Using BestKeeper, standard deviation (SD) and coefficient of variation (CV) values were computed for the reference genes, where lower SD and CV values indicate higher stability. In yellow *S. rugosoannulata*, *VMA* and *MRP* were identified as the most stable genes, while *RPB2* exhibited the least stability. In red *S. rugosoannulata*, *MRP* and *GAPDH* were identified as the most stable genes. Across different colors of the pileus, the lowest SD value was observed for *MRP* and *UBP*, while *RPB2* consistently exhibited the least stable (Fig 3).

Given the variations in stability rankings across different softwares, RefFinder was utilized to provide a comprehensive ranking by calculating the geometric mean of stability weights from geNorm, NormFinder, and BestKeeper. The integrated analysis confirmed *UBP* as the most stable gene in both red and yellow *S. rugosoannulata*, with *MTC* being the most stable in different colors of the pileus (Table 2).

**Table 2:** Ranking of candidate reference genes by RefFinder.

Rank	Red		Yellow		Color of pileus	
	Gene	Geomean	Gene	Geomean	Gene	Geomean
1	UBP	2.11	UBP	2.30	MTC	1.41
2	MRP	2.82	CKA1	2.45	UBP	1.86
3	MTC	2.91	MTC	2.63	ACTIN	2.91
4	VMA	3.25	UBQ	2.91	MRP	2.99
5	RPB2	4.86	VMA	3.83	UBQ	4.82
6	UBQ	5.89	ACTIN	5.00	CKA1	5.48
7	GAPDH	6.18	MRP	5.12	VMA	7.00
8	LEO	6.24	LEO	8.00	LEO1	8.00
9	ACTIN	6.62	GAPDH	9.00	GAPDH	9.00
10	CKA1	7.4	RPB2	10.00	RPB2	10.00

### Optimal number of reference genes for normalization

**Fig 4:**
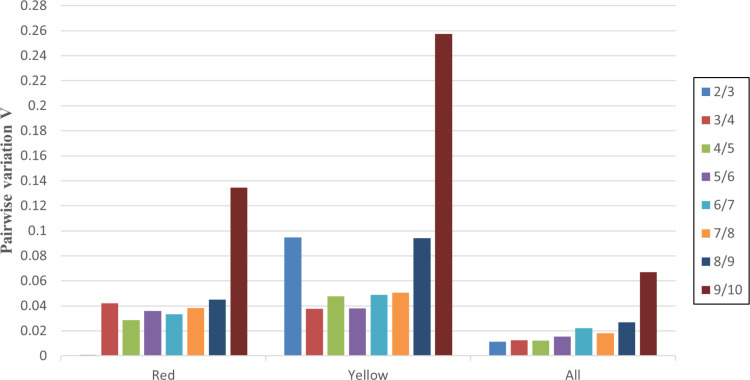
Determination of the optimal number of reference genes for normalization by pairwise variation using geNorm. The cut off value is 0.150, below which the inclusion of an additional reference gene is not required.

To ascertain the optimal number of reference genes for normalization, geNorm calculated pairwise variation (Vn/Vn + 1), employing a cut-off of 0.15, below this level was not required to adding an additional reference gene [36]. All computed V values fell below this cut-off, indicating that two stable reference genes suffice for normalizing gene expression data. The optimal combination of the two most stable genes across all groups is adequate in Fig 4.

### Validation of the best and least stable reference genes

**Fig 5:**
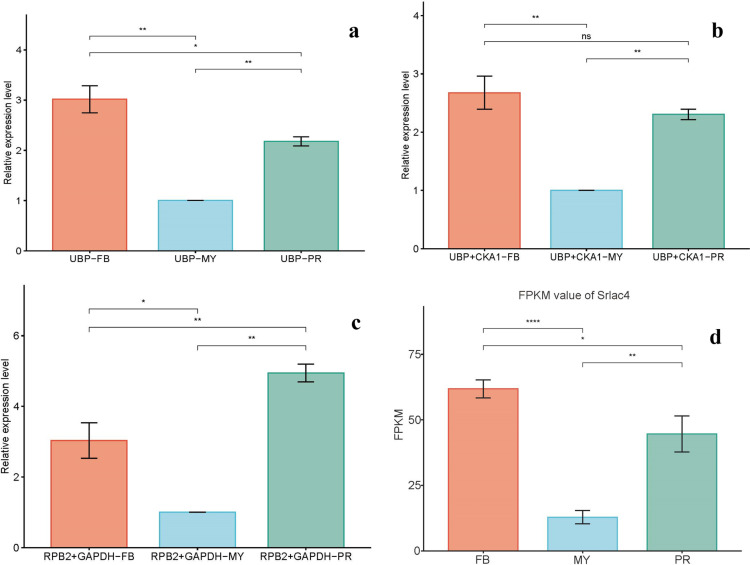
Validation of reference gene quality. Relative *Srlac4* expression levels were normalized using candidate reference genes under different developmental stages of yellow *S. rugosoannulata*.MY: mycelium; PR: primordial; FB: fruiting bodies. a: The results were corrected using the most stable reference gene UBP;b: The results were corrected using the most stable reference gene (UBP and CKA1);c: The results were corrected using the least stable reference gene (RPB2 and GAPDH);d: FPKM values of srlac4 gene transcriptome sequenced in different developmental stages of yellow S. rugosoannulata. Data are displayed as mean ± standard error of the mean. Statistical analyses were performed using Student’s t-test to compare two reference genes or combinations of reference genes for normalization. * P < 0.05; **P < 0.01; ns.: no signifcant diference.

To validate the efficacy of the selected candidate reference genes for normalization, the expression levels of *Srlac4* (laccases) were assessed, which are crucial for phenolic compounds degradation, across different developmental stages [37]. Based on the comprehensive ranking for yellow *S. rugosoannulata*, *UBP* and *CKA1* were chosen as suitable reference genes for expression analysis. In contrast, *RPB2*, the least stable gene, was used to illustrate the impact of selecting an inappropriate reference gene. Normalizing Srlac4 expression against *UBP* and *CKA1* or *UBP* alone indicated higher expression levels in the fruiting body compared to the mycelium and primordium stages. When *RPB2* was used for normalization, the relative expression of *Srlac4* peaked in the primordium. Notably, similar expression patterns for *Srlac4* emerged when utilizing stable reference genes for normalization alongside mRNA-sequencing data from the three developmental stages of yellow *S. rugosoannulata* (Fig 5).

## Discussion

Quantitative reverse transcription-polymerase chain reaction (qRT-PCR) is widely utilized for high-throughput gene transcription analysis due to its enhanced sensitivity and specificity. However, there is no universally applicable reference gene across all species or within different tissues and cells of the same species. For instance, research on *V. volvacea* indicates that *MSF1* exhibits stable expression under cold and high-temperature stress, *UBQ* gene maintains stability under NaCl and CuSO₄ stress, and *MAPK* is most stable under acidic conditions [20]. Similarly, within different strains of *S. rugosoannulata*, the stability of reference genes varies significantly; for example, *CKA1* displays the least stability during various developmental stages of red *S. rugosoannulata* but performs better in yellow strains. This highlights the necessity of selecting appropriate reference genes tailored to specific experimental designs and species [38,22].

Current studies on internal reference gene screening in mushroom species predominantly focus on traditional genes such as 18S ribosomal RNA (*18S*), 28S ribosomal RNA (*28S*), β-actin (*ACTB*), cyclophilin (*CYP*), glyceraldehyde-3-phosphate dehydrogenase (*GAPDH*), and ubiquitin (*UBQ*) [12,19,25]. The instability of these traditional genes can constrain experimental design and treatment applicability. With the advancement of omics sequencing technologies, numerous investigations have emerged that propose reference gene screening based on transcriptomic data in both animals and plants [26,27]. This approach has also shown effectiveness and accuracy in mushroom species [19,29].

In our investigation, we systematically assessed the stability of ten candidate reference genes utilizing four software. Our findings revealed that novel reference genes identified through transcriptomic data exhibited higher stability across various developmental stages of red and yellow *S. rugosoannulata* compared to four traditional reference genes, which exhibited lower stability. Six novel reference genes (*UBP*, *MRP*, *MTC*, *VMA*, *LEO1*, *CKA1*) were identified as reliable for gene expression analysis. Consistent conclusions were drawn from studies involving *V. volvacea* [19], *P. ostreatus* [38], and *Morchella* [29], with one study on *V. volvacea* noting that the increasing availability of RNA-Seq and other expression datasets will facilitate the discovery of additional reference genes with suitable expression levels, underscoring the importance of incorporating this methodology in reference gene selection [39]. These results indicate that reference genes screened based on transcriptomic data display enhanced stability compared to traditional options, and the exploration of novel reference genes can yield more reliable alternatives for ensuring gene expression stability.

In the various developmental stages of yellow *S. rugosoannulata*, the rankings generated by NormFinder and geNorm were notably similar, whereas BestKeeper exhibited greater variability, consistent with previous observations [40–43]. This divergence may stem from the fact that geNorm and NormFinder employ analogous methodologies for stability calculations, while BestKeeper utilizes coefficient of variation (CV) ± standard deviation (SD) for ranking. Given the diverse stability and expression levels of candidate reference genes, it is prudent to integrate stability and expression analyses using different software packages.

To ascertain the optimal number of reference genes, “pairwise variation (V)” was computed using geNorm. A threshold V score of 0.15 was established following the manufacturer’s guidelines [36]. In our study, all V scores across the different developmental stages of red and yellow *S. rugosoannulata* were below 0.15, indicating that two stable reference genes suffice for reliable qRT-PCR normalization.

For evaluating the stability of the selected reference genes, we employed the most stable combination (*UBP* + *CKA1*) alongside the least stable combination (*RPB2* + *GAPDH*) to normalize *Srlac4* expression in yellow *S. rugosoannulata*. The results indicated that normalization with *UBP* + *CKA1* revealed the highest relative expression of *Srlac4* in the fruiting body, followed by the primordium, and the lowest in mycelium, aligning with transcriptome sequencing data. Conversely, normalization using the *RPB2* + *GAPDH* combination showed a markedly different expression pattern, suggesting that employing reference genes with poor stability for qRT-PCR correction may result in erroneous interpretations.

In summary, six novel candidate reference genes have been introduced, with several (*UBP*, *MTC*, *CKA1*) exhibiting ideal stability. This research provides a valuable set of reference genes for different developmental stages of red and yellow *S. rugosoannulata*, offering effective tools for gene expression analysis. In addition, this research also provides guidelines for achieving enhanced accuracy in RT-qPCR analyses for other mushrooms.

## Supporting information

S1 Table Ranking of the candidate reference genes for red *S. rugosoannulata* according to geNorm BestKeeper and NormFinder. (XLS)

S2 Table Ranking of the candidate reference genes for yellow *S. rugosoannulata* according to geNorm NormFinder and BestKeeper. (XLS)

S3 Table Ranking of the candidate reference genes for different colors of the pileus according to geNorm NormFinder and BestKeeper. (XLS)
